# Patient-Reported Outcomes of Arsenic-Related Skin Lesions in China

**DOI:** 10.1155/2020/6195975

**Published:** 2020-09-17

**Authors:** Yajia Li, Danrong Jing, Yi Xiao, Xiaoyan Huang, Minxue Shen

**Affiliations:** ^1^Department of Dermatology, Xiangya Hospital, Central South University, Changsha, China; ^2^Hunan Engineering Research Center of Skin Health and Disease, Central South University, Changsha, China; ^3^Hunan Key Laboratory of Skin Cancer and Psoriasis, Central South University, Changsha, Hunan, China; ^4^Department of Pathology, Xiangya Hospital, Central South University, Changsha, China; ^5^Department of Social Medicine and Health Management, Xiangya School of Public Health, Central South University, Changsha, China

## Abstract

**Purpose:**

Previous studies confirmed that chronic arsenic exposure could lead to pigmentary changes and hyperkeratosis. However, skin health-related quality of life (HRQoL) among people under lifetime arsenic exposure remains underappreciated. Our study is aimed at investigating several patient-reported outcomes in a population under chronic arsenic exposure. *Patients and Methods*. A cross-sectional study was conducted in communities in Shimen, China. Dermatologists performed skin examinations for participants. Patient-reported outcomes (PROs) included HRQoL, itch, sleep quality, and symptoms of anxiety and depression. The Dermatology Life Quality Index (DLQI) was used to measure skin HRQoL. The numerical rating scale (NRS) was used to measure the intensity of itching. Sleep disturbance was measured by Pittsburgh Sleep Quality Index (PSQI). Anxiety and depression were measured by two-item Generalized Anxiety Disorder (GAD-2) and Patient Health Questionnaire (PHQ-2), respectively.

**Results:**

A total of 464 participants suffering from arsenic-related skin lesions finished the assessment of DLQI. Pigmentary changes and arsenical keratosis were not associated with the patient-reported outcomes except PHQ-2. Hair arsenic exceeding 1 *μ*g/g was associated with higher itch NRS and DLQI (*P* < 0.05). Itch NRS (adjusted *β* = 0.80, 95% CI: 0.70–0.90, *P* < 0.01) and hair arsenic concentration (adjusted *β* = 0.12, 95% CI: 0.01–0.24, *P* < 0.05) were independently associated with the DLQI.

**Conclusion:**

HRQoL, sleep quality, and mental wellbeing are impaired in residents under chronic arsenic exposure. Itching and hair arsenic are independent risk factors for impaired HRQoL.

## 1. Introduction

In the study of the global burden of diseases, disfigurement and cutaneous symptoms (itch and pain) are used to determine the disability weights of skin diseases [1]. While skin symptoms cause unpleasant feelings and impair quality of life, the impact of disfigurement varies across cultures and regions. In some specific settings such as regions of arsenicosis, how skin lesions and symptoms affect the quality of life remains underappreciated.

Epidemiologic research on arsenic exposure and its health effects on population boomed worldwide in the last decades [2]. Arsenic ingestion causes characteristic pigmentary changes in the skin of the trunk and extremities and nodular keratosis on the palms and soles. Arsenic can also exert a latent effect to cause skin and internal cancers [2]. Skin lesions were reported as the first sign to indicate arseniasis [3]. Evidence from the United States, India, Bangladesh, Nepal, Chile, and Taiwan confirmed the dose-response relationship between drinking water arsenic concentrations and skin lesions [4–7]. In addition to skin symptoms, pruritus has also been considered an adverse drug reaction in patients receiving arsenic trioxide therapy [8, 9]. However, health-related quality of life (HRQoL), an important aspect of health outcomes, has been rarely reported among residents suffering from arsenicosis.

Mines were extensively exploited in the realgar-rich region in Shimen county from the 1950s and were completely shut down by the government in 2011 [10]. Despite the environmental improvements, arsenic-related health outcomes in the residents continue to occur. Approximately 70% of the local residents have arsenic-related skin lesions (ArSL), and over 50% are suffering from chronic generalized pruritus [11]. Previously, we assessed the psychometric properties of the Dermatology Life Quality Index (DLQI) among ArSL patients [10]. The current study is aimed at investigating patient-reported outcomes (PROs) including itching, sleep quality, and symptoms of anxiety and depression in the residents with the exposure of chronic arsenic.

## 2. Material and Methods

### 2.1. Study Design

This was a cross-sectional study. Three sites near the mining area in Shimen Hunan province, including the Realgar Plant Community, the Heshan Village, and the Wangyangqiao Village, were investigated. We recruited the participants by the health examination which was supported by the Shimen government. Participants with ArSL were included in the current analysis. This study was conducted according to the guidelines laid down in the Declaration of Helsinki. All procedures involving patients were approved by the institutional research ethics boards of Xiangya Hospital, Central South University (approval #201603172). Written informed consent was obtained from all participants.

### 2.2. Diagnosis and Measurements

Certified dermatologists performed skin examinations. ArSL (including pigmentary changes and hyperkeratosis) and other skin diseases were diagnosed mainly according to the cutaneous symptoms, and sometimes skin biopsy and dermatoscopy were used for necessary disease information.

A face-to-face questionnaire survey was conducted in order to inquire about demographic information, the history of exposure to occupational arsenic, the history of medications and diseases, and the characteristics of bath and skincare behaviors.

Hair samples were saved in envelopes and transferred to Shimen Center for Disease Control and Prevention for measurement every day. Hair was washed, treated with mixed acid, and digested overnight. Hair arsenic concentration was determined by nondispersive atomic fluorescence spectrometry (Ruiguang RGF-7800, China). Hair arsenic was then categorized by 1 *μ*g/g according to the World Health Organization's recommendation [12].

### 2.3. Measurement of PROs

Dermatology Life Quality Index (DLQI), a tool for measuring dermatology-specific HRQoL according to the patient-reported outcome (http://www.dermatology.org.uk/), was used to measure the skin health-related quality of life in this study [13]. And it has also been validated in many skin diseases and translated into different languages [14]. The psychometric properties of the DLQI have been assessed in this population [10].

The numerical rating scale (NRS) grading from 0 to 10 was used to measure the current intensity of pruritus. Sleep quality was measured by the Pittsburgh Sleep Quality Index (PSQI) [15]. This measure includes seven subscales: subjective sleep quality, sleep latency (time to fall asleep), sleep duration, habitual sleep efficiency (time asleep/time in bed), sleep disturbance, use of sleeping medications, and daytime dysfunction. Symptoms of anxiety and depression were measured by the two-item Generalized Anxiety Disorder Scale (GAD-2) [16] and two-item Patient Health Questionnaire (PHQ-2) [17], respectively. All the tools were permitted for use in noncommercial research without changes or modifications.

### 2.4. Statistical Analyses

Characteristics of the participants were described as mean ± standard deviation for continuous data and count (percentage) for categorical data. One-way analysis of variance (ANOVA) and chi-square test were used for hypothesis tests according to the type of data. Spearman's correlation coefficients between the patient-reported measures were estimated. A two-level linear model was used to estimate the effect sizes of factors related to DLQI, with participants as level one units and community as level two units. For all statistical tests, 0.05 was regarded as the significance level. Statistical analysis was performed in SAS 9.2 (SAS Institute Inc., Cary, USA).

## 3. Results

There were 771 of 1092 participants who were diagnosed with ArSL, and 464 (60%) of them finished the assessment of DLQI completely. Participants who declined the DLQI evaluation had a significantly higher educational level (*P* = 0.002).

The characteristics of the participants by locations are shown in Table 1. The proportions of female (93%), widowhood (28%), and history of occupational exposure (51%) in the Realgar Plant Community were significantly higher than the other locations. Hair arsenic concentration showed an inverse association with the distance to the previous mining area (Wangyangqiao Village > Heshan Village > Realgar Plant Community), although the differences were not significant. By contrast, the prevalence of pigmentary changes (90%) and hyperkeratosis (29%) in the Realgar Plant Community was not higher than the prevalence in Heshan Village (94% and 31%, respectively). Residents in the Realgar Plant Community had on average the highest level of itch intensity, sleep disturbance, symptoms of anxiety and depression, and impaired skin health-related quality of life measured by the DLQI.

The associations of the DLQI with the intensity of itching, sleep disturbance, and symptoms of anxiety and depression are shown in Table 2. The DLQI score showed a strong positive association with itch NRS (Spearman's *r* = 0.71). By contrast, the associations of the DLQI with other patient-reported outcomes were weak but significant (Spearman's *r* < 0.2, *P* < 0.05). As shown in Table 3, comparisons of PROs across the presence of pigmentary changes or hyperkeratosis showed no significant results except PHQ-2. Hair arsenic exceeding 1 *μ*g/g was associated with higher itch NRS and DLQI (*P* < 0.05).

Risk factors of the DLQI were analyzed in two-level linear models. The intracluster correlation coefficient of the null model was 8.5% and was not statistically significant (*P* = 0.13). According to Table 4, hair arsenic and itch NRS were independent risk factors of DLQI after adjustments for demographic and clinical characteristics of the participants. One unit increase in hair arsenic (*μ*g/g) and itch NRS were associated with 0.12 (95% CI: 0.01–0.24, *P* < 0.05) and 0.8 (95% CI: 0.70–0.90, *P* < 0.01) increase in the DLQI, respectively. Pigmentary changes and hyperkeratosis were not significantly associated with the DLQI.

## 4. Discussion

The study is aimed at investigating HRQoL and mental wellbeing in a population under lifetime arsenic exposure. HRQoL was moderately impaired in residents with ArSL. Itching was a significant risk factor for impaired skin HRQoL. Hair arsenic concentration also shows a positive association with the quality of life after adjustments. However, disfigurement such as pigmentary changes and hyperkeratosis were not associated with HRQoL or other PROs in this population. Sleep disturbance and emotional problems are also presented in addition to disabilities measured by the DLQI.

Among the three locations, the Realgar Plant Community was characterized by owing the highest proportions of female participants, widowhood, and history of occupational exposure to arsenic. Many of the male residents in this community had died from arsenic-related diseases; however, the prevalence of arsenical keratosis among the survivors was not higher than that in Heshan Village, possibly owing to the survivor bias. Despite the effect, this group of participants still had the highest severity of itching, sleep disturbance, symptoms of anxiety and depression, and impaired HRQoL.

The strong association of itch intensity and the DLQI is understandable because the first item of the DLQI (symptoms) was frequently endorsed by most patients. The previous study has shown that itching is an extensive symptom of arsenic exposure [8], and itch significantly affects sleep, daily life, and farm work while scratching and the hot bath are used to alleviate the itching among the local residents. By contrast, disfigurement including pigmentary changes and arsenical keratosis was not likely to evoke stigmatized feelings according to our in-depth interview. In this context, ArSL would probably not affect their interpersonal, sexual, and social activities. This is consistent with the DLQI item distributions and the result of regression for the DLQI.

Although few studies have introduced the main limitations of the DLQI, it is continuously used because of the convenience of application [14].Besides, since it mainly focuses on disabilities, we measured the emotional problems using the GAD-2 and PHQ-2. The presence of skin lesions is associated with irritability and depression can be explained by a local superstition that ArSL was an ominous sign of the following cancers. This speculation can be further validated by the in-depth interview as well as the result that residents with hyperkeratosis had a significantly higher level of PHQ-2.

A primary limitation of the study is that the population is idiosyncratic and the conclusions are more likely to lack generalizability. There may also be selection bias, as participants who refused the DLQI assessments tended to be more educated than those only with a general educational level. Nevertheless, to our knowledge, this is the first study to investigate the HRQoL among the population under lifetime arsenic exposure and among a group of skin-disease patients free from concerns about disfigurement-related stigmatization.

## 5. Conclusion

In summary, impaired quality of life and mental wellbeing were identified in people with ArSL. Itching is an independent risk factor for impaired quality of life, sleep disturbance, and emotional problems. Furthermore, in order to improve sleep and life quality, strategies for itch relief may be essential for the management of patients with ArSL.

## Figures and Tables

**Table 1:** haracteristics of the participants by location^a^.

	Realgar Plant Community	Heshan Village	Wangyangqiao Village	*P*
*N*	59 (13)	297 (64)	108 (23)	
Age (year)	61.7 ± 10.4	60.3 ± 9.4	61.7 ± 9.0	0.429
Gender				
Male	4 (7)	140 (47)	64 (59)	<0.001
Female	55 (93)	157 (53)	44 (41)	
Ethnicity				
Han	30 (51)	206 (69)	93 (86)	<0.001
Other	29 (49)	91 (31)	15 (14)	
Marriage				
Married	42 (72)	246 (83)	97 (91)	0.038
Widowed	16 (28)	41 (14)	7 (6)	
Divorced/unmarried	0 (0)	10 (3)	3 (3)	
Educational level				
Primary school and below	28 (49)	191 (65)	62 (59)	0.051
Middle school	15 (26)	74 (25)	29 (28)	
High school	13 (23)	26 (9)	11 (11)	
College and above	1 (2)	3 (1)	3 (3)	
Household annual income (CNY)				
<10000	29 (51)	152 (52)	47 (44)	0.047
10000–29999	23 (40)	127 (43)	42 (39)	
30000–49999	4 (7)	10 (3)	12 (11)	
≥50000	1 (2)	4 (1)	6 (6)	
Occupational exposure history	30 (51)	41 (14)	4 (4)	<0.001
Hair arsenic (*μ*g/g)	1.6 ± 3.5	1.0 ± 2.5	0.8 ± 1.5	0.310
Arsenic-related skin lesions				
Pigmentary changes	53 (90)	278 (94)	97 (90)	0.344
Hyperkeratosis	17 (29)	91 (31)	23 (21)	0.181
Itch NRS (0–10)	5.9 ± 2.7	5.3 ± 3.2	3.4 ± 3.2	<0.001
PSQI (0–21)	6.8 ± 3.2	5.4 ± 4.0	4.9 ± 3.7	0.006
GAD-2 (0–6)	3.5 ± 1.3	3.2 ± 1.1	3.0 ± 1.1	<0.001
PHQ-2 (0–6)	3.5 ± 1.3	3.1 ± 1.2	2.9 ± 1.1	0.016
DLQI (0–30)	6.0 ± 3.6	5.1 ± 4.1	2.9 ± 3.4	<0.001

^a^BMI: body mass index; IQR: interquartile range; NRS: numeric rating scale; PSQI: Pittsburgh Sleep Quality Index; GAD-2: two-item Anxiety Disorder Scale; PHQ-2: two-item Patient Health Questionnaire; DLQI: Dermatology Life Quality Index. Continuous data are expressed as mean ± standard deviation. Categorical data are expressed as count (%).

**Table 2:** Spearman correlation between the patient-reported measures.

	Itching NRS	PSQI	GAD-2	PHQ-2
PSQI	0.18^∗∗^	1		
GAD-2	0.11^∗^	0.28^∗∗^	1	
PHQ-2	0.13^∗∗^	0.31^∗∗^	0.74^∗∗^	1
DLQI	0.71^∗∗^	0.18^∗∗^	0.17^∗∗^	0.18^∗∗^

NRS: numeric rating scale; PSQI: Pittsburgh Sleep Quality Index; GAD-2: two-item Anxiety Disorder Scale; PHQ-2: two-item Patient Health Questionnaire; DLQI: Dermatology Life Quality Index. ^∗^*P* < 0.05 and ^∗∗^*P* < 0.01.

**Table 3:** Comparisons of patient-reported outcomes by pigmentary changes and hyperkeratosis.

	Pigmentary changes			Hyperkeratosis			Hair arsenic concentration		
PROs	Presence	Absence	*P*	Presence	Absence	*P*	≥1 *μ*g/g	<1 *μ*g/g	*P*
Itch NRS	5.0 ± 3.2	5.0 ± 3.0	0.947	5.4 ± 3.3	4.9 ± 3.3	0.208	5.9 ± 3.2	5.0 ± 3.3	0.012
PSQI	5.5 ± 3.8	4.7 ± 3.9	0.223	5.3 ± 3.9	5.5 ± 3.8	0.555	5.4 ± 4.2	5.4 ± 3.7	0.910
GAD-2	3.2 ± 1.2	3.2 ± 1.1	0.899	3.1 ± 1.0	3.2 ± 1.2	0.129	3.3 ± 1.1	3.2 ± 1.2	0.315
PHQ-2	3.1 ± 1.2	3.1 ± 1.1	0.887	2.9 ± 1.0	3.2 ± 1.2	0.010	3.2 ± 1.2	3.1 ± 1.2	0.522
DLQI	4.6 ± 4.0	5.6 ± 4.1	0.181	5.1 ± 4.0	4.6 ± 4.0	0.211	5.8 ± 4.2	4.5 ± 4.0	0.003

PROs: patient-reported outcomes; NRS: numeric rating scale; PSQI: Pittsburgh Sleep Quality Index; GAD-2: two-item Anxiety Disorder Scale; PHQ-2: two-item Patient Health Questionnaire; DLQI: Dermatology Life Quality Index.

**Table 4:** Factors associated with the DLQI.

	Crude model		Adjusted model	
Characteristics	*β* (95% CI)	*P*	*β* (95% CI)	*P*
Age (year)	-0.03 (-0.08, 0.01)	0.129	-0.03 (-0.07, 0.02)	0.216
Gender (male vs. female)	-0.43 (-1.24, 0.39)	0.305	-0.28 (-0.93, 0.36)	0.389
Race (Han vs. other)	0.73 (-0.15, 1.60)	0.103	-0.06 (-0.73, 0.62)	0.871
Marriage (widowed vs. married)	0.31 (-0.86, 1.48)	0.601	0.10 (-0.84, 1.05)	0.834
Educational level	-0.19 (00.73, 0.34)	0.475	-0.15 (-0.61, 0.32)	0.545
Household annual income	-0.40 (-0.94, 0.13)	0.139	-0.14 (-0.57, 0.29)	0.529
Occupational exposure history	1.21 (0.10, 2.31)	0.032	0.33 (-0.54, 1.19)	0.462
Hair arsenic (*μ*g/g)	0.22 (0.07, 0.38)	0.005	0.12 (0.01, 0.24)	0.047
Pigmentary changes	0.48 (-1.01, 1.97)	0.527	-0.62 (-1.77, 0.52)	0.284
Hyperkeratosis	0.50 (-0.40, 1.39)	0.275	0.13 (-0.55, 0.82)	0.709
Itch NRS	0.84 (0.74, 0.93)	<0.001	0.80 (0.70,0.90)	<0.001
PSQI	0.18 (0.07, 0.28)	0.001	0.03 (-0.05, 0.11)	0.464
GAD-2	0.43 (0.08, 0.78)	0.016	0.01 (-0.41, 0.43)	0.977
PHQ-2	0.43 (0.09, 0.77)	0.014	0.10 (-0.32, 0.52)	0.635

NRS: numeric rating scale; PSQI: Pittsburgh Sleep Quality Index; GAD-2: two-item Anxiety Disorder Scale; PHQ-2: two-item Patient Health Questionnaire; DLQI: Dermatology Life Quality Index; CI: confidence interval.

## Data Availability

Data generated or analyzed during this study are included in this published article. The datasets generated during and/or analyzed during the current study are available from the corresponding author on reasonable request.

## Abbreviations

PROs: Patient-reported outcomes
HRQoL: Skin health-related quality of life
DLQI: Dermatology Life Quality Index
NRS: Numerical rating scale
PSQI: Pittsburgh Sleep Quality Index
GAD-2: Two-item Generalized Anxiety Disorder
PHQ-2: Two-item Patient Health Questionnaire.

## References

[1] Karimkhani C., Dellavalle R. P., Coffeng L. E. (2017). Global skin disease morbidity and mortality: an update from the global burden of disease study 2013. *JAMA Dermatology*.

[2] Smith A. H., Steinmaus C. M. (2009). Health effects of arsenic and chromium in drinking water: recent human findings. *Annual Review of Public Health*.

[3] Ahsan H., Steinmaus C. (2013). Invited commentary: use of arsenical skin lesions to predict risk of internal cancer: implications for prevention and future research. *American Journal of Epidemiology*.

[4] Ahsan H., Chen Y., Parvez F. (2006). Arsenic exposure from drinking water and risk of premalignant skin lesions in Bangladesh: baseline results from the health effects of arsenic longitudinal study. *American Journal of Epidemiology*.

[5] Ghosh P., Banerjee M., de Chaudhuri S. (2007). Comparison of health effects between individuals with and without skin lesions in the population exposed to arsenic through drinking water in West Bengal, India. *Journal of Exposure Science & Environmental Epidemiology*.

[6] Maharjan M., Ahmad S. K. A., Ohtsuka R., Watanabe C. (2005). Arsenic contamination in drinking water and skin manifestations in lowland Nepal: the first community-based survey. *The American Journal of Tropical Medicine and Hygiene*.

[7] Tseng W. P., Chu H. M., How S. W., Fong J. M., Lin C. S., Yeh S. (1968). Prevalence of skin cancer in an endemic area of chronic arsenicism in Taiwan. *Journal of the National Cancer Institute*.

[8] Ohnishi K., Yoshida H., Shigeno K. (2002). Arsenic trioxide therapy for relapsed or refractory Japanese patients with acute promyelocytic leukemia: need for careful electrocardiogram monitoring. *Leukemia*.

[9] Sanaat Z., Rezazadeh M., Gharamaleki J. V., Ziae J. E., Esfahani A. (2011). Arsenic trioxide in patients with refractory multiple myeloma: a prospective, phase II, single-arm study. *Acta Medica Iranica*.

[10] Xiao Y., Huang X., Jing D. (2018). Assessment of the Dermatology Life Quality Index (DLQI) in a homogeneous population under lifetime arsenic exposure. *Quality of Life Research*.

[11] Xiao Y., Huang X. Y., Shen M. X., Zhang J. L., Chen X. (2018). Elevated prevalence of pruritus after life-time arsenic exposure: the baseline survey of a prospective cohort study in Shimen, China. *Journal of the American Academy of Dermatology*.

[12] WHO (1981). *Environmental health criteria 18 - Arsenic*.

[13] Finlay A. Y., Khan G. K. (1994). Dermatology Life Quality Index (DLQI)—a simple practical measure for routine clinical use. *Clinical and Experimental Dermatology*.

[14] Both H., Essink-Bot M. L., Busschbach J., Nijsten T. (2007). Critical review of generic and dermatology-specific health-related quality of life instruments. *The Journal of Investigative Dermatology*.

[15] Buysse D. J., Reynolds C. F., Monk T. H., Berman S. R., Kupfer D. J. (1989). The Pittsburgh Sleep Quality Index: a new instrument for psychiatric practice and research. *Psychiatry Research*.

[16] Kroenke K., Spitzer R. L., Williams J. B. W., Monahan P. O., Löwe B. (2007). Anxiety disorders in primary care: prevalence, impairment, comorbidity, and detection. *Annals of Internal Medicine*.

[17] Kroenke K., Spitzer R. L., Williams J. B. W. (2003). The Patient Health Questionnaire-2: validity of a two-item depression screener. *Medical Care*.

